# Effect of continuity of care on medication adherence and psychological outcomes in patients with coronary heart disease: a retrospective cohort study

**DOI:** 10.3389/fpsyt.2026.1784201

**Published:** 2026-03-13

**Authors:** Juan Wei, Baiwenxian Li, Shaojie Han, Hongjie Zhuang, Wenhong Cao, Hongyan Zhang

**Affiliations:** 1 Department of Cardiology, The First Affiliated Hospital of Hebei North College, Zhangjiakou, Hebei, China; 2 Department of Psychology, The First Affiliated Hospital of Hebei North College, Zhangjiakou, Hebei, China

**Keywords:** anxiety, continuity of care, coronary heart disease, depression, medication adherence, propensity score matching, retrospective cohort study

## Abstract

**Background:**

Coronary heart disease (CHD) is a prevalent cardiovascular disorder worldwide. Post-discharge medication non-adherence and high anxiety/depression rates often lead to disease recurrence and reduced quality of life in CHD patients. While continuity of care is hypothesized to improve these outcomes, existing evidence is limited by high heterogeneity and lack of large-sample standardized validation in CHD populations.

**Objective:**

This study aimed to assess the effects of continuity of care on medication adherence, psychological status, and cardiovascular readmission rates in post-discharge CHD patients.

**Methods:**

A total of 320 CHD patients discharged between January 2021 and June 2023 were divided into an intervention group (structured continuity of care) and a conventional care group. We adopted 1:1 propensity score matching to control baseline confounders, with 128 patients per group included in the final analysis. The primary outcome was 6-month medication adherence measured by Medication Possession Ratio (MPR). Secondary outcomes included Hospital Anxiety and Depression Scale (HADS) scores and cardiovascular readmission rates.

**Results:**

Post-matching, the two groups were well-balanced in baseline characteristics. The intervention group showed significantly higher 6-month MPR (86.3 ± 9.7% vs. 67.5 ± 11.3%; MD = 18.8%, 95% CI: 16.1%–21.5%) and a greater proportion of good adherence (82.0% vs. 53.9%; RR = 1.52, 95% CI: 1.31–1.77). HADS anxiety and depression scores were notably lower in the intervention group (anxiety: 6.1 ± 2.2 vs. 9.0 ± 2.6; depression: 5.7 ± 2.4 vs. 8.6 ± 2.8). The 6-month cardiovascular readmission rate was significantly reduced in the intervention group (5.5% vs. 16.4%; RR = 0.33, 95% CI: 0.15–0.72).

**Conclusion:**

Continuity of care is associated with improved long-term medication adherence, alleviated anxiety and depression, and reduced cardiovascular readmission risk in post-discharge CHD patients. It is recommended to gradually incorporate it into the routine management of cardiovascular disease rehabilitation after its efficacy has been validated in multicenter studies.

## 1 Introduction

Coronary heart disease is a cardiovascular disease caused by coronary atherosclerosis leading to vascular stenosis or occlusion and myocardial ischemia and hypoxia. Its incidence and mortality rate remain high worldwide, making it a major public health problem (1).1. The long-term rehabilitation management of CHD patients has been a clinical focus since the late 20th century (2). With the advancement of medical technology, the success rate of acute phase treatment for CHD patients has significantly improved, but long-term rehabilitation management after discharge is still the key to improving prognosis (3).

Clinical practice has found that patients often face two core problems after discharge (4): first, poor medication adherence. Statistics show that the medication adherence rate of CHD patients is less than 70% within 6 months after discharge (5), and irregular medication use directly leads to disease recurrence and increased risk of vascular events; second, prominent psychological problems. The chronic course of the disease itself, treatment pressure, and worries about prognosis result in approximately 30%-40% of CHD patients having comorbid anxiety or depression (6). These psychological comorbidities not only impair individual quality of life but also contribute significantly to the population-level mental health burden associated with chronic cardiovascular conditions, driving increased healthcare utilization and compounding public health costs 66. Negative emotions further reduce medication adherence, forming a vicious cycle of “disease-psychology-adherence”.

As a patient-centered care model, continuity of care emphasizes continuous and personalized health management from hospitalization to post-discharge family and community. Its core contents include discharge plan connection, continuous health monitoring, medication guidance, psychological support, and health education (7). From a public mental health perspective, structured continuity of care represents a scalable, system-level intervention that can be integrated into primary and community care settings to address the mental health needs of chronic disease populations at scale, thereby reducing the overall burden of anxiety and depression in this high-risk group (8). In recent years, the application of continuity of care in chronic disease management has attracted increasing attention. Multiple Meta-analyses and systematic reviews have confirmed the improvement effect of remote intervention and nurse-led care models on anxiety, depression, and medication adherence in CHD patients (9–11). However, existing studies still have problems such as high heterogeneity and non-randomized design in some cases. In addition, the analysis of the synergistic influence mechanism between psychological status and medication adherence is insufficient, and the long-term effectiveness in CHD patients still lacks large-sample evidence-based support (12, 13).

Based on this, this study adopted a retrospective cohort design combined with propensity score matching to control confounding factors, systematically evaluated the effect of continuity of care on medication adherence, anxiety, depression, and readmission rate of CHD patients after discharge, and further explored its potential as a public mental health strategy for chronic disease management. The findings aim to provide scientific basis for optimizing the long-term rehabilitation management strategy of CHD patients and inform health system planning for integrated cardiovascular and mental health care.

## 2 Materials and methods

### 2.1 Study subjects

This study consecutively enrolled patients diagnosed with coronary heart disease (CHD) and discharged from the Department of Cardiology of our hospital between January 2021 and June 2023. Consecutive sampling was adopted to minimize selection bias. The First Affiliated Hospital of Hebei North College is a Grade III Class A general hospital in Zhangjiakou City, Hebei Province, with the Cardiology Department as a key clinical specialty of Hebei Provincial Health Commission. The department has a standardized cardiac rehabilitation center, with professional medical teams including cardiologists, clinical pharmacists, psychologists and dietitians, and has rich experience in the diagnosis, treatment and follow-up management of coronary heart disease patients, which ensures the scientificity and standardization of the study implementation.

Inclusion criteria: (1)) Refer to the “Primary Care Guidelines for Stable Coronary Heart Disease (2018)” (14), confirmed by coronary angiography or CTA; (2) Age ≥18 years old; (3) Stable condition at discharge without serious complications; (4) Complete electronic health records, with accessible pharmacy refill records and follow-up information;(5) Possession of complete pre-discharge baseline HADS score records. Exclusion criteria: (1) Complicated with severe liver and kidney dysfunction, malignant tumors, cognitive impairment and other diseases affecting follow-up; (2) History of mental illness or receiving antipsychotic drug treatment; (3) Unable to cooperate with telephone or online follow-up; (4) Lost to follow-up or incomplete data during the follow-up period;(5) Moderate to severe anxiety or depression (HADS-A or HADS-D ≥ 11 points) was present before discharge.

Ethics statement: This retrospective study was approved by the Ethics Committee of the First Affiliated Hospital of Hebei North University (Approval No.: K2020090). The requirement for informed consent was waived. All procedures adhered to the Declaration of Helsinki. Patient information was de-identified to ensure privacy.

### 2.2 Grouping method and sample size flow

**Figure 1:**
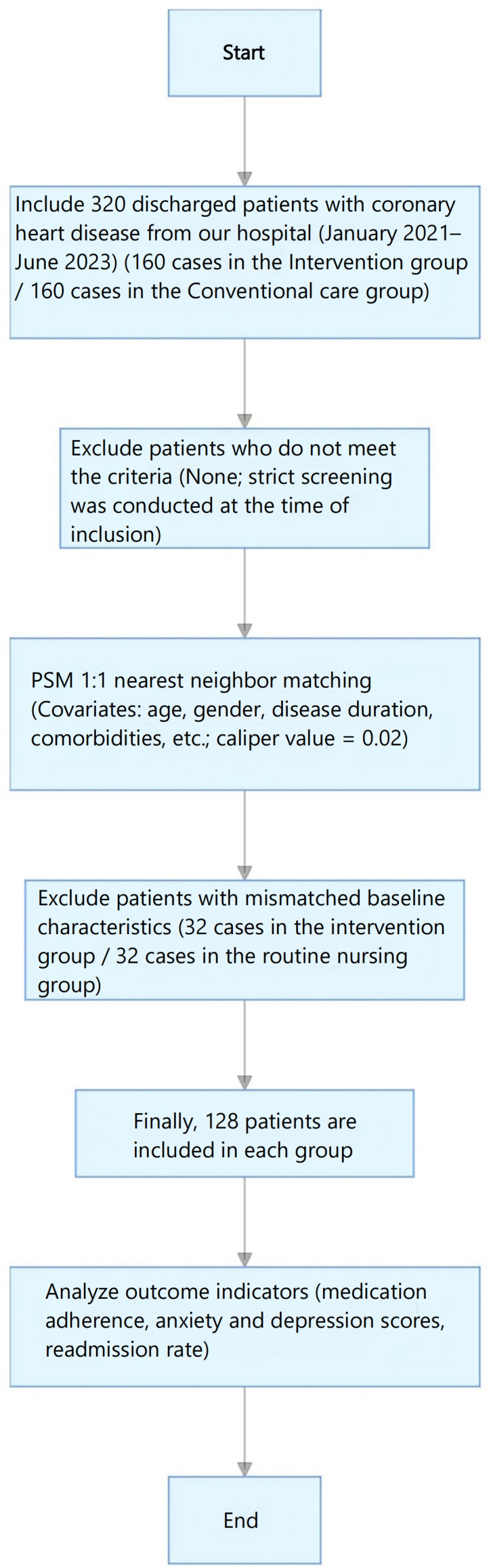
Flow chart of sample size selection.

Patients were divided into two groups based on the hospital’s post-discharge care protocol. The standardized structured continuity of care program was launched in January 2021. Eligible patients agreeing to participate were assigned to the intervention group. Patients discharged before the launch or who declined participation received routine discharge care and were assigned to the conventional care group. To address potential selection bias, propensity score matching (PSM) was used to balance baseline confounding factors between groups (15, 16). Sample size flow (**Figure 1**): Initially, 160 patients per group were enrolled. After 1:1 PSM (nearest neighbor matching, caliper=0.02), 32 patients from each group with unmatched baseline characteristics were excluded. Finally, 128 patients per group were included for analysis.

### 2.3 Nursing measures

#### 2.3.1 Conventional care group

Responsible nurses provided one-time health guidance at discharge, covering CHD knowledge, medication precautions, diet (low salt/fat), exercise suggestions, and review schedule. An outpatient contact number was provided. No active follow-up was conducted post-discharge.

#### 2.3.2 Intervention group

In addition to conventional care, patients received a 6-month structured continuity of care program, developed based on the Guidelines for Cardiac Rehabilitation of Coronary Heart Disease Patients in China (2021 Edition) and validated transitional care frameworks (17). To ensure intervention standardization, the following fidelity measures were implemented:

1. Staff training: All nurses and doctors involved in the intervention received 20 hours of standardized training, covering discharge plan formulation, follow-up communication skills, HADS scale assessment, and medication guidance (18).
2. Consistency monitoring: A special quality control team conducted monthly checks on 20% of the follow-up records to verify the completeness and compliance of intervention implementation; non-compliant cases were fed back to the responsible staff for rectification (19).
3. Standard operating procedures (SOPs): A detailed SOP manual was formulated for each intervention link to ensure consistent implementation across different staff members (20).

The core components of the continuity of care program are summarized in **Supplementary Table 1**.

### 2.4 Outcome indicators

(1) Primary outcome indicator: Medication adherence

Evaluated by Medication Possession Ratio (MPR). Calculation method: (Total days of actual medication possession by patients within 6 months ÷ Total theoretical days of medication needed) ×100%. MPR≥80% was defined as good medication adherence (21).MPR was calculated for all prescribed cardiovascular medications, including antiplatelet drugs, statins, beta-blockers, and angiotensin-converting enzyme inhibitors/angiotensin receptor blockers (22).In addition to MPR assessment, all irregular medication-taking behaviors of patients during the 6-month follow-up period were systematically documented through telephone follow-up and medical record review, including four categories: inaccurate dosage (taking more/less than the prescribed dose), incorrect timing (missing the prescribed administration time or taking at irregular intervals), self-discontinuation (stopping medication without the doctor’s approval), and self-switching (changing the type of medication without the doctor’s approval). For patients with irregular medication behaviors, the research team recorded the specific type, frequency and causes of the behavior in a unified case report form, and conducted targeted intervention in time (including re-education on medication knowledge, adjustment of medication regimen, and setting up medication reminders). In the statistical analysis, the correlation between the type/frequency of irregular medication behaviors and MPR was further analyzed, and the influence of irregular medication on the final medication adherence evaluation was corrected to improve the accuracy of the assessment results.

(2) Secondary outcome indicators:

1. Anxiety and depression: Evaluated by the HADS scale, which includes two subscales: Anxiety (HADS-A) and Depression (HADS-D). Each subscale contains 7 items, each item is scored 0–3 points, with a total score of 0–21 points. A higher score indicates more severe anxiety or depression; The Chinese version of the HADS scale was used in this study, which has been validated for reliability and validity in Chinese cardiovascular disease populations (Cronbach’s α coefficient = 0.82-0.86) (23, 24). Outcome assessment was performed by two independent professional raters, including trained nurses from the hospital’s nursing quality control department (excluded from the study’s intervention implementation team) and clinical psychologists from the Department of Psychology (2). To minimize detection bias, a partial blinding method was adopted: the raters were blinded to the patients’ original group allocation (intervention group/conventional care group) and the research purpose of the study, and only received the basic information of the patients (excluding care intervention-related information) and the unified assessment training. The two raters conducted independent double assessment for all patients, and the kappa coefficient was used to test the inter-rater reliability (kappa > 0.85, P < 0.001), ensuring the consistency of the assessment results. If there was a discrepancy between the two assessment results, a third senior psychologist would conduct a joint review to determine the final score.
2. Cardiovascular Readmission Rate: Counted readmissions caused by CHD-related events such as angina pectoris, myocardial infarction, and heart failure within 6 months. Data were extracted from the hospital’s electronic medical record system by independent research staff blinded to group allocation.

### 2.5 Statistical methods

Statistical analysis was performed using SPSS 26.0 and R 4.1.3 software. Categorical data were expressed as case numbers (percentage) [n (%)], with intergroup comparisons conducted using χ² test or Fisher’s exact probability method. For continuous data, normality testing was performed first; if normal, the data were presented as mean ± standard deviation (x ± s), with intergroup comparisons using t-test. If non-normal, the data were expressed as median (interquartile range) [M (P25, P75)], with intergroup comparisons using Mann-Whitney U test. propensity score matching (PSM) was employed to balance baseline characteristics between the two groups, with continuous care as the dependent variable. Factors potentially influencing outcomes, such as gender, age, disease duration, baseline HADS-A score, and baseline HADS-D score, were included as covariates. The nearest neighbor matching method was used, with a caliper value of 0.02 and a matching ratio of 1:1, to calculate propensity scores and perform matching. After PSM matching, the baseline characteristics of the two groups achieved good balance (all SMDs <0.1), and intergroup differences were negligible. Therefore, independent samples t-test and χ² test were used for comparison, a method widely applied in matched studies of the same type (15). Pearson correlation analysis was used to explore the relationship between medication adherence (MPR value) and anxiety and depression scores. A P-value <0.05 was considered statistically significant.

## 3 Results

### 3.1 Comparison of baseline characteristics between the two groups after PSM

After PSM matching, there were no statistically significant differences in baseline characteristics such as age, gender, disease duration, comorbidities (hypertension, diabetes mellitus, hyperlipidemia), educational level, marital status, and family care status between the two groups (all SMD<0.1, P>0.05), indicating good comparability (**Table 1**). The achievement of baseline balance through PSM strengthens the internal validity of the study and provides a more robust foundation for comparing subsequent outcome measures between groups.

**Table 1:** Comparison of baseline characteristics between the two groups after PSM (n=128).

Baseline characteristics	Intervention group	Conventional care group	t/χ² value	P value	SMD
Age (years), ( $\bar{\text{x}}$ ± s)	65.3 ± 8.2	64.7 ± 7.9	0.532	0.595	0.07
Gender (male), n (%)	76 (59.4)	73 (57.0)	0.158	0.691	0.05
Disease duration (years), ( $\bar{\text{x}}$ ± s)	5.2 ± 2.1	5.4 ± 2.3	0.674	0.501	0.09
Complicated with hypertension, n (%)	92 (71.9)	89 (69.5)	0.213	0.644	0.06
Complicated with diabetes mellitus, n (%)	58 (45.3)	55 (42.9)	0.147	0.702	0.05
Complicated with hyperlipidemia, n (%)	85 (66.4)	82 (64.1)	0.176	0.675	0.05
Educational level (high school and above), n (%)	53 (41.4)	50 (39.1)	0.152	0.696	0.05
Marital status (married), n (%)	106 (82.8)	103 (80.5)	0.287	0.592	0.06
With family caregivers, n (%)	98 (76.6)	95 (74.2)	0.235	0.628	0.06
Health literacy (medium and above), n (%)	68 (53.1)	65 (50.8)	0.121	0.728	0.04
Social support (medium and above), n (%)	75 (58.6)	72 (56.3)	0.105	0.768	0.04

### 3.2 Comparison of medication adherence between the two groups

The medication possession ratio and the rate of good medication adherence in the intervention group within 6 months after discharge were significantly higher than those in the conventional care group, with statistically significant differences (both P<0.001) (**Table 2**). The absolute improvement in MPR (18.8%) and the increased likelihood of good adherence (RR = 1.52) suggest that continuity of care is associated with a clinically meaningful enhancement in medication-taking behavior. From a clinical perspective, an MPR increase of over 15% is considered sufficient to improve the control of cardiovascular risk factors such as blood lipid and blood pressure, thereby reducing the incidence of adverse cardiovascular events. In addition, the correlation analysis between irregular medication behaviors and MPR showed that the total frequency of irregular medication behaviors was moderately negatively correlated with MPR in the whole cohort (r=-0.526, P<0.001). Among all types of irregular medication behaviors, self-discontinuation (r=-0.481, P<0.001) and inaccurate dosage (r=-0.395, P<0.001) had the strongest negative correlation with MPR. After correcting the influence of irregular medication behaviors on the medication adherence evaluation, the MPR of the intervention group (85.7 ± 10.2%) was still significantly higher than that of the conventional care group (66.2 ± 11.8%) (t=11.935, P<0.001), and the proportion of good medication adherence in the intervention group (80.5%) remained significantly higher than that in the conventional care group (51.6%) (χ²=23.842, P<0.001), which further confirmed the improvement effect of continuity of care on medication adherence. The incidence of all types of irregular medication behaviors was significantly lower in the intervention group than in the conventional care group (all P<0.05), with the most significant difference in self-discontinuation behavior (10.2% vs. 35.9%, P<0.001).

**Table 2:** Comparison of medication adherence between the two groups within 6 months after discharge.

Group	Number of cases (n)	Medication possession ratio (%, mean ± SD)	t	P	Good medication adherence n(%) (MPR≥80%)	χ²	P	RR (95%CI)	MPR after correction (%, mean ± SD)	t	P	Good adherence after correction n(%)	χ²	P
Intervention group	128	86.3 ± 9.7	12.846	<0.001	82.0%	26.734	<0.001	1.52 (1.31-1.77)	85.7 ± 10.2	11.935	<0.001	80.5%	23.842	<0.001
Conventional care group	128	67.5 ± 11.3	–	–	53.9%	–	–	–	66.2 ± 11.8	–	–	51.6%		

### 3.3 Comparison of HADS scale scores between the two groups

The HADS-A score and HADS-D score of the intervention group within 6 months after discharge were significantly lower than those of the conventional care group, with statistically significant differences (both P<0.001) (**Table 3**). The observed reductions in both anxiety and depression scores (approximately 2.9 points for each) exceed the threshold for the minimal clinically important difference (MCID) for the HADS in cardiac populations, which is typically around 1.5 points. This indicates that the improvement in psychological symptoms associated with continuity of care is likely to be perceptible and relevant to patients’ lived experience. In clinical practice, a reduction of HADS score by more than 2 points is usually associated with improved sleep quality, enhanced treatment compliance, and better quality of life in CHD patients.

**Table 3:** Comparison of HADS scale scores between the two groups within 6 months after discharge (points, mean ± SD).

Scale dimension	Intervention group (n=128)	Conventional care group (n=128)	t value	P value	MD (95%CI)	Inter-rater kappa
Anxiety subscale (HADS-A)	6.1 ± 2.2	9.0 ± 2.6	9.352	<0.001	-2.9 (-3.5 to -2.3)	0.88
Depression subscale (HADS-D)	5.7 ± 2.4	8.6 ± 2.8	8.764	<0.001	-2.9 (-3.6 to -2.2)	0.88

### 3.4 Comparison of readmission rate due to cardiovascular events between the two groups

There were 7 readmissions related to cardiovascular events in the intervention group within 6 months, with a readmission rate of 5.5%; 21 readmissions in the conventional care group, with a readmission rate of 16.4%. The readmission rate of the intervention group was significantly lower than that of the conventional care group, with a statistically significant difference (χ²=8.927, P = 0.003; RR = 0.33, 95% CI: 0.15-0.72) (**Table 4**). This represents a substantial relative risk reduction. However, given the observational nature of this study, this finding should be interpreted as a strong association rather than definitive proof of causality, as residual confounding cannot be fully ruled out.

**Table 4:** Comparison of readmission rate due to cardiovascular events between the two groups within 6 months after discharge.

Group	Number of cases (n)	Readmitted (n)	Not readmitted (n)	Readmission rate (%)	χ² value	P value	RR (95%CI)
Intervention group	128	7	121	5.5%	8.927	0.003	0.33 (0.15-0.72)
Conventional care group	128	21	107	16.4%	–	–	–

### 3.5 Correlation between medication adherence and psychological status

To explore the internal connection between key outcome indicators, Pearson correlation analysis was performed between MPR and HADS scores of all 256 patients. The results showed that medication possession ratio (MPR) was moderately negatively correlated with HADS anxiety score (r=-0.402, P<0.001) and depression score (r=-0.385, P<0.001). This exploratory analysis provides preliminary evidence for an interrelationship between medication adherence and psychological well-being in this cohort, supporting the conceptual link between these domains.

Potential mechanisms underlying this correlation may include two aspects. First, alleviated anxiety and depression can reduce patients’ negative emotions about treatment, thereby improving their initiative to adhere to medication. Second, good medication adherence helps stabilize the patient’s condition, which in turn reduces the psychological burden caused by disease recurrence or deterioration, forming a positive feedback loop. It is important to note that this correlation does not imply causation and may be influenced by shared underlying factors such as social support level and health literacy.

## 4 Discussion

This study adopted a retrospective cohort design combined with propensity score matching, effectively controlling for multiple baseline confounding factors (all SMD < 0.1 after matching). The results showed that coronary heart disease patients who received structured continuous care had significantly higher medication adherence 6 months after discharge, milder symptoms of anxiety and depression, and a lower risk of cardiovascular-related readmission. These findings are consistent with previous evidence on the beneficial effects of continuous care (5, 13). It should be emphasized that, given the non-randomized observational design of this study, the above results should be interpreted as strong associations rather than conclusive causal relationships. Nevertheless, these associative findings provide an important scientific reference for optimizing long-term rehabilitation management strategies for coronary heart disease patients and suggest their potential public health value in alleviating the mental health burden associated with chronic comorbidities (25).

### 4.1 Promotion of medication adherence and self-management capacity and its clinical significance

Medication adherence is the cornerstone of long-term secondary prevention for coronary heart disease (CHD). In this study, the continuous care group, through a closed-loop management model of “assessment-guidance-monitoring-intervention”, achieved an absolute increase of 18.8 percentage points in medication possession ratio (MPR) and a 1.52-fold relative increase in the proportion of good adherence. This magnitude surpasses the effects reported in many medication adherence interventions from clinical trials, implying that it may translate into better control of low-density lipoprotein cholesterol and blood pressure, as well as a lower risk of vascular events in real-world settings (26). The core lies in the systematic support that enhanced patients’ self-management capacity, which is consistent with findings from studies on transitional care improving self-care levels in elderly patients undergoing percutaneous coronary intervention (PCI) (18). Regular follow-up not only addressed practical barriers to medication use (e.g., doubts, concerns about adverse reactions) but also transformed passive medication-taking into active health management behaviors through continuous education and feedback, aligning with the mechanism by which structured psychosocial interventions indirectly improve adherence by reinforcing health awareness (12).

### 4.2 Alleviation of anxiety and depressive symptoms: from individual perception to public health implications

This study observed an average reduction of approximately 2.9 points in HADS anxiety and depression scores in the continuous care group. This reduction is not only highly statistically significant but, more importantly, exceeds the widely recognized minimal clinically important difference threshold (approximately 1.5 points) for this scale in cardiac patient populations (27). This indicates that the intervention-induced symptom relief has clear clinical relevance and is likely perceived by patients themselves, improving their disease experience and quality of life.

Placing this finding in a broader context, the 30%–40% comorbidity rate of anxiety and depression among CHD patients constitutes a heavy burden of individual suffering and public health, leading to increased disability, rising medical costs, and elevated mortality (28, 29). Therefore, the implications of this study extend beyond the assessment of a single clinical intervention effect. It demonstrates that a relatively standardized, nurse-led continuous care model can effectively identify and alleviate psychological distress in routine cardiovascular rehabilitation. From an integrated perspective of public mental health and chronic disease management, such a model represents a scalable, “targeted prevention” strategy for a known high-risk population (30). Nuño et al. (2012) proposed the ICCC Framework, emphasizing that integrated care for chronic conditions should integrate medical care, psychological support, and community management, which provides a theoretical basis for the multi-dimensional intervention of continuity of care in this study (7). If successfully integrated into regional primary health care networks or chronic disease management systems, it is expected to reduce the burden of mental comorbidities in the CHD population at the systemic level, break the cycle of mutual deterioration between “mind and body”, and thereby generate extensive public health benefits by reducing overall medical costs and improving population health outcomes (31).

### 4.3 Impact on readmission risk: association, pathways, and cautious interpretation

Readmission is a key hard endpoint for evaluating healthcare quality and prognosis. This study shows that continuous care was associated with a 67% reduction in the risk of cardiovascular-related readmission within 6 months (RR = 0.33). We propose a plausible mediating pathway hypothesis for future validation: continuous care may jointly improve physiological index stability and symptom control through two core pathways—enhancing medication adherence and alleviating psychological distress—ultimately leading to a reduction in readmission risk. This hypothesis is supported by the literature; for example, a study by Pogosova et al. confirmed that anxiety and depression are independent predictors of adverse outcomes (32).

However, this significant association must be interpreted with caution. Although multiple confounding factors were controlled through propensity score matching (PSM), the inherent limitations of a retrospective observational design mean that unmeasured factors (e.g., subtle differences in disease severity, strength of social support networks, health literacy) may still partially explain the result (33). Therefore, this should be regarded as strong, suggestive evidence of a potential causal relationship, rather than conclusive proof. The more important value of this result lies in providing a compelling rationale and key effect size reference for subsequent prospective studies, especially randomized controlled trials focusing on health economic evaluation.

### 4.4 Limitations, and future directions

#### 4.4.1 Limitations

1) Fundamental design constraints: The retrospective, non-randomized design carries risks of residual confounding and selection bias, which is the primary limitation; 2) Follow-up duration: The 6-month follow-up is insufficient to assess long-term effects and the natural trajectory of psychological symptoms (34); 3) Measurement limitations: MPR is not completely equivalent to actual medication intake; the single-center design limits generalizability; 4) Lack of economic evidence: This study did not collect cost data or conduct a formal cost-effectiveness analysis, and the mentioned health economic potential is only an inference based on clinical outcomes.5) Blinding limitation: Due to the characteristics of the retrospective cohort study and the non-pharmacological intervention of continuity of care, the intervention implementers could not be blinded to the group allocation of patients. However, the outcome assessment was completed by independent raters who were blinded to the group allocation and research purpose, and strict inter-rater reliability verification was adopted, which effectively reduced the detection bias caused by non-blinding and ensured the objectivity of the outcome assessment results.

#### 4.4.2 Future directions

1) Conducting multicenter prospective randomized controlled trials to provide high-level causal evidence; 2) Designing studies with longer follow-up periods (≥2 years) to evaluate sustained effects; 3) Embedding health economic evaluations in intervention studies to systematically collect cost and health utility data; 4) Exploring the integration of digital health tools (e.g., mobile applications) to improve intervention accessibility, personalization, and efficiency, while paying attention to differences in technology acceptance to ensure equity; 5) Within an implementation science framework, investigating how to integrate and adapt such effective models into different healthcare systems and community settings, with a particular focus on coverage for resource-limited or underserved populations to promote health equity. Exploring stratified promotion strategies: In resource-abundant regions, promote the multidisciplinary continuous care model of “nurse + nutritionist + psychologist”; in primary or resource-limited areas, simplify intervention procedures by leveraging rural doctors and community nurses to conduct telephone follow-ups and basic medication guidance, while utilizing free online platforms to disseminate health education content, thereby narrowing the urban-rural care gap (35).

## 5 Conclusion

This study demonstrates that structured continuity of care is significantly associated with improved 6-month medication adherence, reduced anxiety and depression symptoms, and lower cardiovascular readmission risk in post-discharge coronary heart disease (CHD) patients. These findings provide real-world evidence for optimizing long-term CHD rehabilitation management and support the integration of continuity of care into clinical practice. Given the retrospective observational design, the above associations should not be interpreted as definitive causal relationships. Further validation of the long-term effectiveness and cost-effectiveness of this care model is required through multicenter, prospective randomized controlled trials in diverse healthcare settings and patient populations.

## Data Availability

The raw data supporting the conclusions of this article will be made available by the authors, without undue reservation.
