# Supplementary material for: Effect of continuity of care on medication adherence and psychological outcomes in patients with coronary heart disease: a retrospective cohort study
Source: Front Psychiatry. 2026 Mar 13;17:1784201. doi: 10.3389/fpsyt.2026.1784201 (PMC13021638; doi:10.3389/fpsyt.2026.1784201)
Supplement: Supplementary file 1 [file Table1.docx]

Table S1 Core Components of the Structured Continuity of Care Program

| **Core Measure** | **Implementation Frequency** | **Implementation Method** | **Responsible Personnel** | **Key Content** |
| --- | --- | --- | --- | --- |
| Discharge Plan Formulation | 3 days before discharge | Multidisciplinary team discussion | Cardiologist, responsible nurse, dietitian | Evaluate patient’s condition, medication status, psychological status and family care ability; formulate personalized follow-up, review and emergency plans |
| Regular Follow-up | 1 week after discharge,then once a month for 6 times | Telephone/WeChat video | Responsible nurse | Monitor medication status, symptom changes, lifestyle implementation and psychological status; record and solve problems in a timely manner |
| Medication Review | Included in each follow-up | Pharmacy record review + patient interview | Pharmacist, responsible nurse | Check medication list; adjust guidance according to condition changes; intervene for patients with poor adherence (e.g., set medication reminders) |
| Psychological Assessment and Intervention | At discharge, 3rd month and 6th month of follow-up | HADS scale assessment + targeted counseling | Clinical psychologist, responsible nurse | Screen for anxiety/depression (score ≥8 points as abnormal); provide counseling for abnormal cases; refer to psychology department if necessary |
| Health Education | Phased education during follow-up | Online articles, video tutorials, one-on-one explanation | Medical team | Disease rehabilitation knowledge, medication importance, emotional management skills, emergency treatment methods |
